# Automated SNOMED CT concept and attribute relationship detection through a web-based implementation of cTAKES

**DOI:** 10.1186/s13326-019-0207-3

**Published:** 2019-09-18

**Authors:** Martijn G. Kersloot, Francis Lau, Ameen Abu-Hanna, Derk L. Arts, Ronald Cornet

**Affiliations:** 1 Department of Medical Informatics, Amsterdam Public Health Research Institute, Amsterdam UMC, University of Amsterdam, Meibergdreef 9, 1105AZ Amsterdam, The Netherlands; 2 School of Health Information Science, University of Victoria, Victoria, Canada

**Keywords:** Chart abstraction, Natural language processing, Electronic health records, Algorithms, SNOMED CT

## Abstract

**Background:**

Information in Electronic Health Records is largely stored as unstructured free text. Natural language processing (NLP), or Medical Language Processing (MLP) in medicine, aims at extracting structured information from free text, and is less expensive and time-consuming than manual extraction. However, most algorithms in MLP are institution-specific or address only one clinical need, and thus cannot be broadly applied. In addition, most MLP systems do not detect concepts in misspelled text and cannot detect attribute relationships between concepts. The objective of this study was to develop and evaluate an MLP application that includes generic algorithms for the detection of (misspelled) concepts and of attribute relationships between them.

**Methods:**

An implementation of the MLP system cTAKES, called DIRECT, was developed with generic SNOMED CT concept filter, concept relationship detection, and attribute relationship detection algorithms and a custom dictionary. Four implementations of cTAKES were evaluated by comparing 98 manually annotated oncology charts with the output of DIRECT. The F_1_-score was determined for named-entity recognition and attribute relationship detection for the concepts ‘lung cancer’, ‘non-small cell lung cancer’, and ‘recurrence’. The performance of the four implementations was compared with a two-tailed permutation test.

**Results:**

DIRECT detected lung cancer and non-small cell lung cancer concepts with F_1_-scores between 0.828 and 0.947 and between 0.862 and 0.933, respectively. The concept recurrence was detected with a significantly higher F_1_-score of 0.921, compared to the other implementations, and the relationship between recurrence and lung cancer with an F_1_-score of 0.857. The precision of the detection of lung cancer, non-small cell lung cancer, and recurrence concepts were 1.000, 0.966, and 0.879, compared to precisions of 0.943, 0.967, and 0.000 in the original implementation, respectively.

**Conclusion:**

DIRECT can detect oncology concepts and attribute relationships with high precision and can detect recurrence with significant increase in F_1_-score, compared to the original implementation of cTAKES, due to the usage of a custom dictionary and a generic concept relationship detection algorithm. These concepts and relationships can be used to encode clinical narratives, and can thus substantially reduce manual chart abstraction efforts, saving time for clinicians and researchers.

## Background

Much of the data present in Electronic Health Records (EHRs) are stored as unstructured free text [1] as clinicians often resort to making free-text notes, despite available coding options [2]. The use of free text should be taken into account when EHR data are reused for other purposes [3], since data reuse for research and development of clinical decision support tools can improve healthcare [4]. However, using free-text notes for searching, summarizing, statistical analysis, and as input for decision support systems is challenging [5].

One of the tasks of natural language processing (NLP) methods, named-entity recognition, aims to extract structured information from free text that is less expensive and time-consuming than extracting it manually [6]. NLP in the medical field, medical language processing (MLP), is more challenging than NLP in various other fields since clinical texts have different grammar, contain ambiguous abbreviations (i.e., the same set of letters has multiple meanings), and contain more misspellings [1, 7]. Recent studies show that MLP can successfully be used for several purposes including deriving comorbidities from the EHR [8], detecting adverse events [9], and finding eligible patients for clinical trials by attaching clinical concepts to patient charts (encoding) [10]. Furthermore, MLP has been proven successful in extracting diagnoses from free-text notes from the EHR, thereby reducing manual chart abstraction efforts. It can, for example, be used to automatically detect the recurrence of breast cancer in patient charts, reducing the number of manually reviewed charts by 90% [11]. Other research shows that MLP can identify uncodified diabetes cases, leading to a more complete ascertainment of diagnoses and, thus, better information provision and targeted care for patients [12].

MLP systems include multiple algorithms to process free text and extract information from it. Clinical Text Analysis and Knowledge Extraction System (cTAKES) is an open-source MLP system from The Apache Software Foundation [13]. It is based on the Unstructured Information Management Architecture (UIMA) framework and the OpenNLP toolkit [13]. cTAKES provides linguistic and semantic annotations for unstructured free text [13] using SNOMED CT [14] and RxNorm [15] dictionaries. cTAKES is designed to be modular and extensible at the information model and method levels, ensuring that it is suitable for a variety of use cases [16].

MLP algorithms have been implemented in various systems. A recent systematic review has shown that most implementations of MLP algorithms are institution-specific, address only one clinical need, might be overfitted, and thus not scalable [17]. In addition, most MLP systems do not detect concepts in misspelled text, e.g. ‘Smll cell lng cancer’, only detect unqualified relationships (e.g. Non-small cell lung cancer relates in a way to Recurrent, Fig. 1.1) between concepts or their instances, and cannot detect attribute relationships, e.g. Non-small cell lung cancer with Recurrent as Clinical course (Fig. 1.2). Attribute relationships make the type of relationship between concepts or their instances explicit (e.g. Clinical Course in Fig. 1.2).

**Fig. 1:**
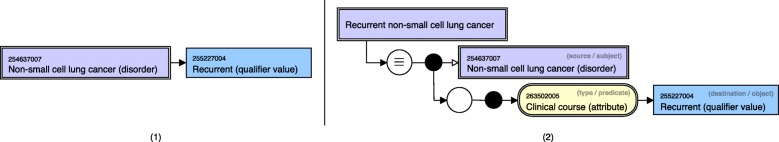
A representation of a unqualified relationship between Non-small cell lung cancer and Recurrent (1) and an attribute relationship of Non-small cell lung cancer with Recurrent as Clinical course (2). The attribute relationship is modelled as a SNOMED CT concept definition diagram of recurrent non-small cell lung cancer (a new post-coordinated expression). Purple blocks represent defined concepts, blue blocks represent primitive SNOMED CT concepts and yellow blocks represent attributes. Attribute groups are represented using a white circle, and conjunctions are represented using a black dot

Since most MLP systems do not offer these algorithms, this study aimed to develop a cTAKES implementation that includes generic algorithms for the detection of concepts from properly spelled and misspelled descriptions and attribute relationships between these concepts. The implementation is evaluated by encoding free-text oncology charts to detect charts that describe recurrent non-small cell lung cancer, and use these outcomes to calculate the F_1_-score.

## Material and methods

### cTAKES

**Fig. 2:**
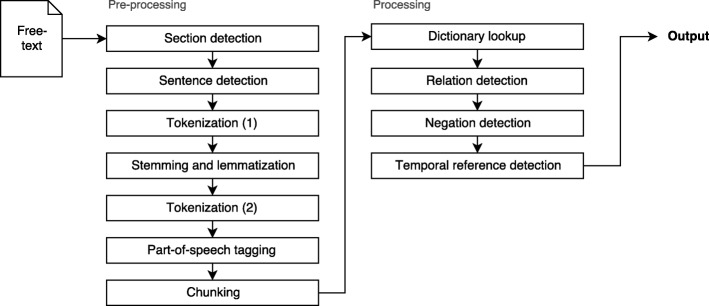
A visual representation of the cTAKES AggregatePlaintextFastUMLSProcessor pipeline

cTAKES enables encoding through several algorithms, which are included in several pipelines. We used cTAKES’ AggregatePlaintextFastUMLSProcessor pipeline (e.g. the output of one algorithm becomes the input to the next [18]), as shown in Fig. 2, for the pre-processing and processing of free-text clinical narratives, since it uses the Unified Medical Language System (UMLS) [19] as its dictionary. In this project, we focus on the SNOMED CT concepts that are included in the UMLS, as the hierarchical and relational structure of SNOMED CT allows us to determine and define relationships between medical concepts.

### Development of an MLP tool

**Fig. 3:**
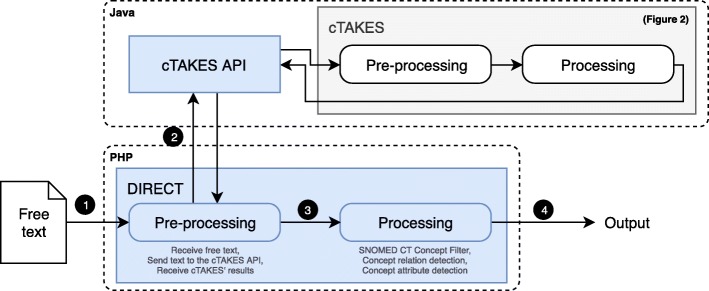
A visual representation of DIRECT in relation to cTAKES. API: Application programming interface. Blue blocks represent developed components, rounded blocks MLP algorithms

Our project involved the development of a cTAKES implementation named Disease Information and Relationship ExtraCtion Tool (DIRECT). cTAKES provides a generic way of concept matching (through dictionary look-up), and detection of syntactic relationships and temporal references. However, it does not detect attribute relationships between concepts (e.g. Small cell carcinoma of lung with Recurrent as Clinical course) and does not match concepts in misspelled text. DIRECT was designed to filter SNOMED CT concepts matched by cTAKES, detect concepts that cTAKES did not detect, and detect attribute relationships between the detected concepts (example shown in Fig. 1.2). Figure 3 shows the workflow of DIRECT. When a user uploads a document or enters free text in the web interface of the tool (Fig. 3.1), DIRECT calls the cTAKES Application Programming Interface (API, Fig. 3.2) to obtain annotations from cTAKES. The detected annotations are processed in DIRECT (Fig. 3.3) and the result is returned to the user (Fig. 3.4).

#### cTAKES API

An API was developed to communicate with cTAKES. It sends text or the contents of a file, received from an HTTP POST request, to the pipeline. The API sets up a UIMA Java environment with cTAKES and the received text and runs the AggregatePlaintextFastUMLSProcessor pipeline. After the pipeline has annotated the text, the results are parsed and returned as an XML file by the API, which is used by the processing algorithm of DIRECT. The XML file contains the detected syntactic relationships and concept identifiers of UMLS concepts, accompanied with the spans of the related terms in the text.

#### DIRECT

DIRECT is a general-purpose web application that allows users to annotate free text originating from a clinical source by sending it to cTAKES using the cTAKES API. Users can focus on the presence of specific concepts (e.g. Primary malignant neoplasm of lung) and their children (e.g. Small cell carcinoma of lung is a Primary malignant neoplasm of lung) or the relationship between concepts, since DIRECT’s algorithms are generic and do not specifically focus on specific concepts. After the text is processed by cTAKES, DIRECT uses algorithms to detect and filter SNOMED CT concepts, detect relationships between concepts, and uses those relationships to detect attribute relationships. These processing algorithms are described below (SNOMED CT concept filter, Concept relationship detection, and Attribute relationship detection). The output of the annotation and the algorithms is formatted and shown to the user.

##### SNOMED CT concept filter

The SNOMED CT concept filter algorithm extracts the most relevant concepts from the output of cTAKES. It derives the SNOMED CT concepts related to the UMLS concept identifier provided by cTAKES and checks the status of every concept (e.g. active if the concept is still in use in SNOMED CT, inactive if not). If the concept is inactive, the filter derives its substitute concepts. Once only active concepts are selected, the algorithm detects if the term listing the concept overlaps with other terms, thus determining if multiple concepts are detected in the same span. In case of an overlap, the algorithm selects the concept associated with the longest term (e.g. ‘Lung cancer’: instead of ‘Lung’). Finally, the algorithm analyzes every span in the text to filter duplicate concepts and to select the most detailed concept in case of a parent-child relation.

##### Concept relationship detection

This algorithm uses cTAKES’ *ConllDependencyNode* identifier to obtain syntactic relationships between words (tokens) that are detected in the text (e.g. in ‘recurrent cancer’, ‘recurrent’ is an adjective that relates to the noun ‘cancer’, Fig. 4.1). The concept relationship detection algorithm matches words from the syntactic relationships (dependencies), to concepts that are detected in the same span. First, the algorithm includes words that have nominal subject (nsubj), adjectival modifier (amod), modifier of nominal (nmod), adverbial modifier (advmod), noun compound modifier (nn), attribute (attr), direct object (dobj), object of a preposition (pobj) and modifier in hyphenation (hmod) relationships (Fig. 4.1) [20]. Next, the algorithm detects if there are concepts detected by cTAKES in the span that the word is part of (‘*recurrent’* in Fig. 4.2). The relationship detection algorithm also tries to match words to SNOMED CT concepts, in case the concept was not detected by the cTAKES NER algorithm, using the same syntactic relationships. It includes free-text descriptions that partly match a SNOMED CT description, calculated by a similarity detection algorithm [21]. Alternative spellings as ‘nonsmall cell lung cancer’ and ‘non small cell lung cancer’ will now map to the description in SNOMED: ‘non-small cell lung cancer’. This similarity detection algorithm is based on PHP’s built-in similar_text function [22]. The algorithm calculates the percentage of similarity by dividing the result of matching characters in two descriptions by the average of the descriptions’ lengths. We included descriptions that matched a concept description for at least 95%, corresponding with a 5% error margin. This error margin did not result in false positive outcomes. Next, the descriptions were matched to the corresponding concept to include SNOMED CT concepts that were misspelled (‘*nonsmall cell lung cancer’* in Fig. 4.2). Once the concepts are detected, the algorithm adds these as origin or destination in the relationship (relationship between ‘*recurrent’* and ‘*Non-small cell lung cancer*’ in Fig. 4.3).

**Fig. 4:**
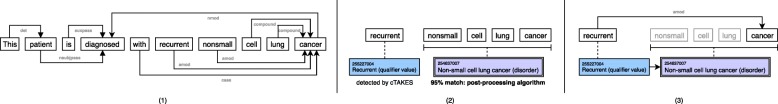
Transformation of the syntactic relationships in the sentence ‘This patient is diagnosed with recurrent nonsmall cell lung cancer’ (1) to a SNOMED CT concept (2) and a relationship between detected SNOMED CT concepts (3, Recurrent and Non-small cell lung cancer)

##### Attribute relationship detection

The algorithm to detect attribute relationships analyzes all relationships and searches a possible attribute relationship between the two concepts by using the SNOMED CT Machine Readable Concept Model (MRCM). The MRCM represents the SNOMED CT concept model rules and it includes domains (e.g. Clinical finding), attributes (e.g. Clinical course), and ranges (e.g. Courses) [23]. The attribute relationship detection algorithm retrieves all possible attribute type SNOMED CT concepts (predicates) that link the destination concept (object) to the source concept (subject) and it adds this attribute concept to the relationship to form a new post-coordinated expression (e.g. Non-small cell lung cancer with Recurrent as Clinical course, Fig. 4).

##### Custom dictionary

Version 2016AB of the UMLS was converted to a cTAKES dictionary using the cTAKES dictionary creator [24]. A custom, plain-text, cTAKES dictionary was developed to link specific keywords that are not included in the UMLS to UMLS concepts. These UMLS concepts are converted to the corresponding SNOMED CT concept in the processing of cTAKES’ output. Words that can be spelled in different ways, such as ‘recurrence’ will now also map to the right concept, such as ‘255227004 | Recurrent (qualifier value) |’.

### Evaluation of the developed MLP tool

To determine which aspect of DIRECT adds value to the annotation of free text, we compared DIRECT to different implementations: cTAKES with the 2011 UMLS version (out-of-the-box, UMLS2011), cTAKES with the 2016AB UMLS version (UMLS2016), and cTAKES with the 2016AB UMLS version and a custom dictionary (UMLS2016 + Dict.). The free-text clinical notes were used as input for each implementation. The output for named-entity recognition and attribute relationship detection was collected and compared to the manual annotation using an R script. Named-entity recognition focused on the detection of SNOMED CT concepts 93880001 | Primary malignant neoplasm of lung (disorder) |, 254637007 | Non-small cell lung cancer (disorder) |, and 255227004 | Recurrent (qualifier value) |. Attribute relationship detection focused on the detection of the SNOMED CT relationship 93880001 | Primary malignant neoplasm of lung (disorder) |: 263502005 | Clinical course (attribute) | = 255227004 | Recurrent (qualifier value) |. This resulted in the number of true positives, false positives, true negatives, and false negatives. These classifications were then used to calculate the precision, recall, and the F_1_-score (Eq. 1, Eq. 2 and Eq. 3, Table 1), the harmonic mean of precision and recall. To compare the performance of the four implementations, a two-tailed permutation test was used, since our dataset is small [25].

Eq. 1 $$ Precision=\frac{TP}{TP+ FP} $$

Eq. 2 $$ Recall=\frac{TP}{TP+ FN} $$

Eq. 3 $$ F1- score=2\cdotp \frac{Precision\cdotp Recall\kern0.5em }{Precision+ Recall} $$

**Table 1:** Variables used in the F-score equation

Algorithm	True positive (TP)	False positive (FP)	False negative (FN)
Named-entity recognition^a^	Same medical concept identified as golden standard.	Identified medical concept differs from golden standard.	Medical concept mentioned, but not identified.
Attribute relationship detection^b^	Attribute relationship present and detected.	Attribute relationship not present, but detected.	Attribute relationship present, but not detected.

^a^SNOMED CT concepts 93880001 | Primary malignant neoplasm of lung (disorder) |, 254637007 | Non-small cell lung cancer (disorder) |, and 255227004 | Recurrent (qualifier value)

^b^SNOMED CT relationship 93880001 | Primary malignant neoplasm of lung (disorder) |: 263502005 | Clinical course (attribute) | = 255227004 | Recurrent (qualifier value)

^c^SNOMED CT concept 255227004 | Recurrent (qualifier value)

#### Used charts

For the evaluation of DIRECT, 98 English (non-small cell) lung cancer patient charts in the form of treatment progress notes from six different centers were included (Table 2). The cases were provided and approved for use by Jonn Wu (JW), an oncologist at BC Cancer, Canada, and manually annotated by a student (Shan Rajapakshe). Both JW and MK determined if the annotations were of good quality. Thirty randomly selected charts are used as development set. The charts are assigned a positive, negative, or not-listed label, based on the occurrence (independent of the number of concept mentions) of the concepts. This label will be leveraged as reference standard in the evaluation process. 50% of the development set and 44% of the test set consist of charts that mention recurrence, either positive or negative.

**Table 2:** Specification of the included charts

Set	Outcome	Lung cancer		Non-small cell lung cancer		Recurrence	Relation
		Implied	Strict	Implied	Strict	Implied	Strict
Development set (*n* = 30)	Positive	27	23	17	17	10	6
	Negative	–	–	–	–	5	–
	Not listed	3	7	13	13	15	13
Test set (*n* = 68)	Positive	51	40	36	31	20	10
	Negative	–	–	–	–	10	–
	Not listed	17	28	32	37	38	58

Relation: Relationship between Lung cancer and Recurrence

Named-entity recognition is evaluated with an implied and a strict approach for the detection of lung cancer and non-small cell lung cancer. The implied approach includes charts that imply that there is an instance of the concept, but do not name the concept, e.g. ‘nonsmall cell’ instead of ‘non-small cell lung cancer’. The strict approach only focuses on the charts that do name the concept.

## Results

### Development of DIRECT

**Fig. 5:**
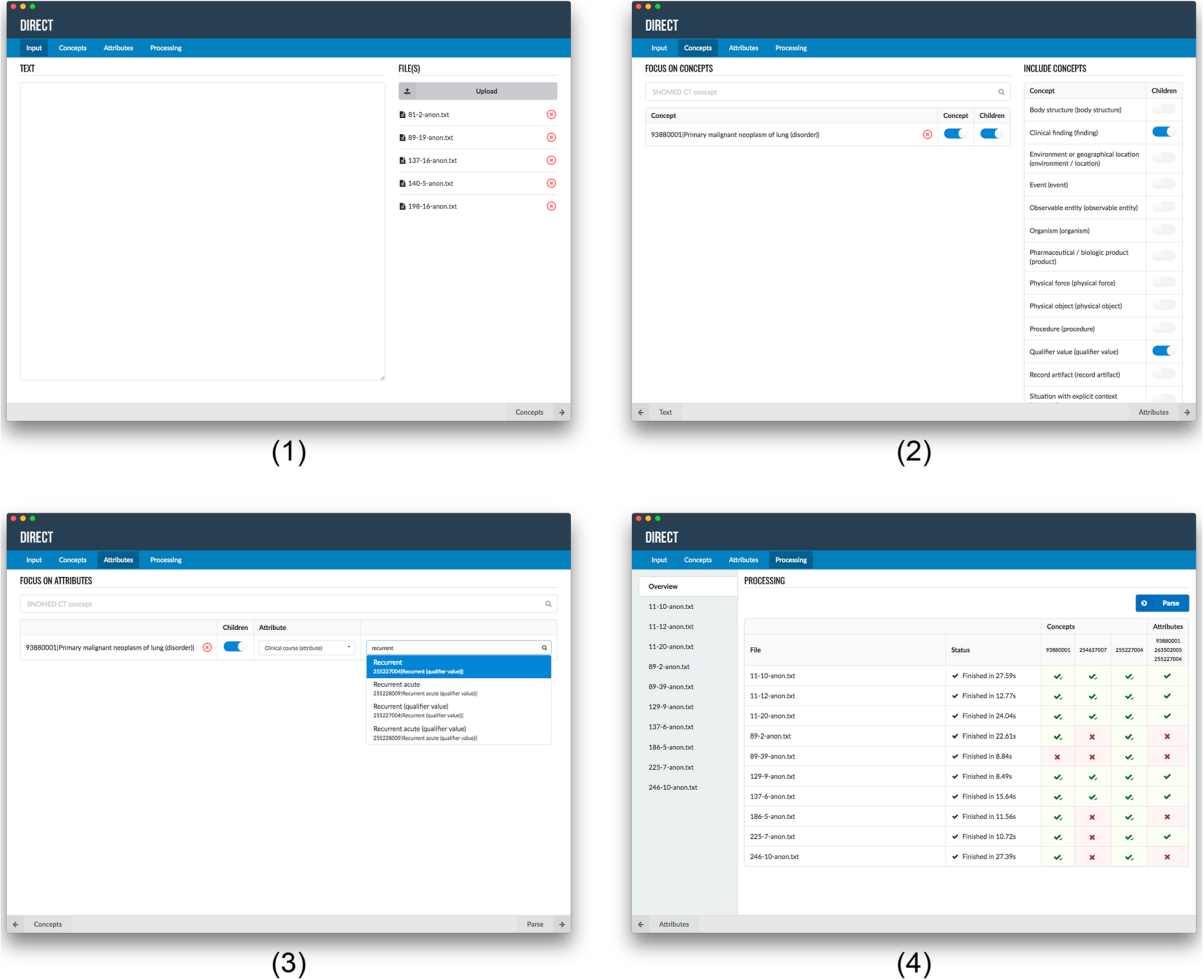
Screenshots of DIRECT. 1. Input free text using a text field or text file(s). 2. Selection of SNOMED CT concepts to focus on and top-level concepts to include**.** 3. Selection of SNOMED CT attribute relationships to focus on. 4. Processing of the free text and results

The concepts were added to the dictionary, according to the methods described. The resulting custom dictionary can be found in Appendix A. DIRECT and the cTAKES API were developed over a period of 4 months by one developer (MK). Figure 5 shows the workflow in the application. Users enter text from a clinical source (Fig. 5.1) and select specific concepts (e.g. Primary malignant neoplasm of lung, Fig. 5.2) and their children, or the relationship between concepts (e.g. Small cell carcinoma of lung with Recurrent as Clinical course, Fig. 5.3) to focus on. The results of the annotation of the free text are then shown to the user (Fig. 5.4).

### Evaluation of the algorithms of DIRECT

Table 3 shows the outcomes of the evaluation of named-entity recognition and attribute relationship detection for the three concepts, the data used for calculating the metrics (true positives, false positives, and false negatives) can be found in Appendix B. The calculated F_1_-scores can also be found in Table 4. The results are shown resp. for the implementations, the implied and strict approach, and the actual presence and absence of the concept in the text. All relationships in the development set were detected, however, not all relationships in the test set were detected. In all but one of the cases, the F_1_-score of DIRECT was higher than the scores of the other implementations, the only exception being non-small cell lung cancer, having an F_1_-score that was higher in the UMLS2011 implementation. The UMLS2016 + Dict. implementation and DIRECT did detect ‘recurrence’, where the UMLS2011 and UMLS2016 implementations did not. DIRECT did not detect recurrence in one case.

**Table 3:** Precision, recall and calculated F-scores from the evaluation outcomes

				Development set (*n* = 30)			Test set (*n* = 68)		
Implementation	Algorithm	Concept	Approach	Precision	Recall	F-score	Precision	Recall	F-score
UMLS2011	Named-entity recognition	Lung cancer^a^	Implied	0.938	0.556	0.698	1.000	0.686	0.814
cTAKES with UMLS 2011			Strict	0.938	0.652	0.769	0.943	0.825	0.880
		Non-small cell lung cancer^b^	Implied	0.917	0.647	0.759	0.967	0.806	0.879
			Strict	0.917	0.647	0.759	0.967	0.935	0.951
		Recurrence^c^		0.000	0.000	0.000	0.000	0.000	0.000
UMLS2016	Named-entity recognition	Lung cancer^a^	Implied	1.000	0.370	0.541	1.000	0.569	0.725
cTAKES with UMLS 2016			Strict	1.000	0.435	0.606	1.000	0.725	0.841
		Non-small cell lung cancer^b^	Implied	1.000	0.294	0.455	0.947	0.500	0.655
			Strict	1.000	0.294	0.455	0.947	0.581	0.720
		Recurrence^c^		0.000	0.000	0.000	0.000	0.000	0.000
UMLS2016 + Dict.	Named-entity recognition	Lung cancer^a^	Implied	1.000	0.852	0.920	1.000	0.706	0.828
cTAKES with UMLS 2016 and custom dictionary			Strict	1.000	1.000	1.000	1.000	0.900	0.947
		Non-small cell lung cancer^b^	Implied	1.000	0.765	0.867	0.957	0.611	0.746
			Strict	1.000	0.765	0.867	0.957	0.710	0.815
		Recurrence^c^		1.000	1.000	1.000	0.882	1.000	0.938
DIRECT	Named-entity recognition	Lung cancer^a^	Implied	1.000	0.852	0.920	1.000	0.706	0.828
cTAKES with UMLS 2016, custom dictionary, and additional processing			Strict	1.000	1.000	1.000	1.000	0.900	0.947
		Non-small cell lung cancer^b^	Implied	1.000	1.000	1.000	0.966	0.778	0.862
			Strict	1.000	1.000	1.000	0.966	0.903	0.933
		Recurrence^c^		1.000	1.000	1.000	0.879	0.967	0.921
	Attribute relationship detection	Recurrent lung cancer^d^		1.000	1.000	1.000	1.000	0.750	0.857

^a^SNOMED CT concept 93880001 | Primary malignant neoplasm of lung (disorder)

^b^SNOMED CT concept 254637007 | Non-small cell lung cancer (disorder)

^c^SNOMED CT concept 255227004 | Recurrent (qualifier value)

^d^Relationship between three SNOMED CT concepts: 93880001 | Primary malignant neoplasm of lung (disorder) |: 263502005 | Clinical course (attribute) | = 255227004 | Recurrent (qualifier value)

**Table 4:** F-scores calculated from the evaluation outcomes

			Development set (*n* = 30)				Test set (*n* = 68)			
Algorithm	Concept	Approach	UMLS2011	UMLS2016	UMLS2016D	DIRECT	UMLS2011	UMLS2016	UMLS2016D	DIRECT
Named-entity recognition	Lung cancer^a^	Implied	0.698	0.541	0.920	0.920	0.814	0.725	0.828	0.828
		Strict	0.769	0.606	1.000	1.000	0.880	0.841	0.947	0.947
	Non-small cell lung cancer^b^	Implied	0.759	0.455	0.867	1.000	0.879	0.655	0.746	0.862
		Strict	0.759	0.455	0.867	1.000	0.951	0.720	0.815	0.933
	Recurrence^c^		0.000	0.000	1.000	1.000	0.000	0.000	0.938	0.921
Relationship detection	Recurrent lung cancer^d^					1.000				0.857

^a^SNOMED CT concept 93880001 | Primary malignant neoplasm of lung (disorder)

^b^SNOMED CT concept 254637007 | Non-small cell lung cancer (disorder)

^c^SNOMED CT concept 255227004 | Recurrent (qualifier value)

^d^Relationship between three SNOMED CT concepts: 93880001 | Primary malignant neoplasm of lung (disorder) |: 263502005 | Clinical course (attribute) | = 255227004 | Recurrent (qualifier value)

The results of the two-tailed permutation test for comparison between the different implementations and DIRECT are shown in Table 5. The test showed statistically significant differences (*P* <  0.05) in outcomes between DIRECT and the UMLS2016 implementation for the detection of lung cancer and non-small cell lung, demonstrating that these differences are not coincidental [25]. There were no significant differences between the detection of lung cancer and non-small cell lung cancer in the original cTAKES (with UMLS 2011) and DIRECT, despite the higher F-scores for the detection of lung cancer. However, there was a significant difference between the detection of recurrence in these implementations.

**Table 5:** Outcomes of the two-tailed permutation test between the different implementations Statistically significant values (*p* <  0.05) are in bold face

Implementation 1	Implementation 2	Lung cancer^a^	Non-small cell lung cancer^b^	Recurrence^c^
UMLS2011	UMLS2016	**0.024**	**0.002**	1.000
UMLS2011	UMLS2016 + Dict.	1.000	**0.016**	**< 0.001**
UMLS2011	DIRECT	1.000	1.000	**< 0.001**
UMLS2016	UMLS2016 + Dict.	**0.024**	0.142	**< 0.001**
UMLS2016	DIRECT	**0.018**	**0.001**	**< 0.001**
UMLS2016 + Dict.	DIRECT	1.000	**0.035**	1.000

^a^SNOMED CT concept 93880001 | Primary malignant neoplasm of lung (disorder)

^b^SNOMED CT concept 254637007 | Non-small cell lung cancer (disorder)

^c^SNOMED CT concept 255227004 | Recurrent (qualifier value)

## Discussion

In this study we implemented DIRECT, a custom version of cTAKES that includes an API and MLP algorithms, and evaluated it using (non-small cell) lung cancer cases. Our results show that DIRECT can identify lung cancer, non-small cell lung cancer and recurrence concepts in charts with precisions of 1.000, 0.966, and 0.879, either with implied and strict approaches, compared to strict precisions of 0.943, 0.967, and 0.000 in the original cTAKES implementation.

Use of either the UMLS2011 or UMLS2016 dictionary results in statistically significant differences in F_1_-scores for named-entity recognition. The old dictionary (2011) consists of all the synonyms of SNOMED CT, NCI Thesaurus, MeSH, and ICD-9 whereas the new dictionary (2016) solely consists of SNOMED CT concepts and descriptions. Therefore, many concepts in the new dictionary have less descriptions and are thus harder to detect using the algorithm. DIRECT does detect these concepts with its algorithms, however with an F_1_-score for the detection of lung cancer that does not differ significantly from that of the UMLS2011 implementation.

The statistically significant differences in F_1_-scores between the UMLS2011 implementation and DIRECT for non-small cell lung cancer can be explained by the relationship detection algorithm. The noun phrase as adverbial modifier relationship (npadvmod) was not included in the relationship detection. One case that contains this relationship between non-small cell and lung cancer could not be detected due to the missing relationship in the algorithm.

One chart included the text ‘recurrent disease’. Since this description is longer than ‘recurrent’, DIRECT chose the SNOMED concept for recurrent disease over the concept for recurrent. This explains why the F_1_-score for the detection of recurrence is higher in the UMLS2016 + Dict. implementation compared to DIRECT.

The relationship ‘lung cancer with recurrent as clinical course’ is detected with an F_1_-score of 0.857. cTAKES does detect relationships between complex adjective and noun combinations (e.g. ‘Recurrent T3 N0 nonsmall cell (adenocarcinoma) lung cancer’ or ‘recurrence of stage IIB non-small lung cancer’ instead of ‘recurrent non-small cell lung cancer’), however it labels them with the wrong syntactic category. Therefore, the algorithm cannot detect the attribute relationship. This problem might be solved in DIRECT by changing the algorithm to include other syntactic relationships as well.

The F_1_-scores show that DIRECT can substantially reduce manual chart abstraction efforts for these concepts. Possible reasons for not tagging concepts are spelling mistakes that are off by more than 5% from the SNOMED CT concept description or concept relationships in texts that are not detected, such as charts that mention cancer in one sentence and specify it as non-small cell in another sentence.

Strengths of our study include the customizability of the dictionaries and the selection of focus concepts in the user interface. This makes DIRECT generic (i.e. non-institution-specific) and allows it to be used for different study designs. Moreover, the algorithms used in DIRECT are generic and therefore not bound to non-small cell lung cancer charts. The algorithms are described in detail, thus these algorithms can be replicated in other implementations. We also used a UMLS dictionary, which is scalable due to the large quantity and variety of concepts available in the UMLS.

Several limitations of our study should be noted. The number of cases is acceptable, but a larger dataset could be used to give the evaluation more power. Additionally, the split-sample evaluation exclusively focussed on (non-small cell) lung cancer with cases that had limited (e.g. ‘nonsmall cell’) to no misspelled concepts and is not externally validated. We did not use cross-validation, since the creation of custom dictionaries and the development of the algorithms based on the development data is highly labour-intensive. Other implementations or cases could give other outcomes, partly due to the non-scalable non-small cell lung cancer custom dictionary.

Comparing the outcome of the algorithms with algorithms found in literature is challenging, since algorithms are often developed for specific implementations and evaluated by encoding specific free-text narratives. We therefore could not compare our outcomes to outcomes described in other MLP papers.

Complete and structured EHR data can improve health care by allowing data to be reused for research and development of clinical decision support tools [4]. This study found that DIRECT can be used to detect specific oncology concepts in free text. We believe that DIRECT and the algorithms described in this paper may be used in other medical settings as well. Clinicians can use DIRECT to get acquainted with MLP, without building their own MLP pipelines. Batches of free text can be processed by the cTAKES API, saving time for clinicians and researchers, who would otherwise have to abstract information manually.

Future studies should validate MLP tools such as cTAKES and DIRECT using cross-validation and external validation and should investigate a different implementation for (non-small cell) lung cancer or other specialisms. Furthermore, the performance of the relationship detection algorithm could be improved and further research is needed to provide methods for that. Future studies should also critically assess the encoded attribute relationships, since DIRECT does not determine if the detected relationships are clinically correct and relevant.

## Conclusion

In this study we developed and evaluated the MLP tool DIRECT, an implementation of cTAKES. We demonstrated how DIRECT could be used to detect oncology concepts through a web interface and how it could detect attribute relationships using MLP algorithms with significant increase in F_1_-score, compared to the original implementation of cTAKES. DIRECT can be used to encode clinical narratives, and thus substantially reduce manual chart abstraction efforts, saving time for clinicians and researchers.

## Data Availability

The datasets used in the current study are not publicly available, for the researchers were granted access to the data for this study only. The source code of DIRECT can be found at https://github.com/martijnkersloot/direct and the R script used for the permutation test can be found at 10.6084/m9.figshare.7539494.

## Appendix

**Table 6:** Contents of the custom dictionary

CUI	Concept description	Type	Type description	Words
C2945760	Recurrent	T079	Temporal Concept	recurrent
C2945760	Recurrent	T079	Temporal Concept	recurring
C2945760	Recurrent	T079	Temporal Concept	recurrence
C0007131	Non-Small Cell Lung Carcinoma	T191	Neoplastic Process	non-small cell lung carcinoma
C0007131	Non-Small Cell Lung Carcinoma	T191	Neoplastic Process	nonsmall cell lung carcinoma
C0007131	Non-Small Cell Lung Carcinoma	T191	Neoplastic Process	non-small cell lung adenocarcinoma
C0007131	Non-Small Cell Lung Carcinoma	T191	Neoplastic Process	nonsmall cell lung adenocarcinoma
C0007131	Non-Small Cell Lung Carcinoma	T191	Neoplastic Process	non-small cell carcinoma of the lung
C0007131	Non-Small Cell Lung Carcinoma	T191	Neoplastic Process	nonsmall cell carcinoma of the lung
C0007131	Non-Small Cell Lung Carcinoma	T191	Neoplastic Process	non-small cell adenocarcinoma of the lung
C0007131	Non-Small Cell Lung Carcinoma	T191	Neoplastic Process	nonsmall cell adenocarcinoma of the lung
C0007131	Non-Small Cell Lung Carcinoma	T191	Neoplastic Process	non small cell lung carcinoma
C0007131	Non-Small Cell Lung Carcinoma	T191	Neoplastic Process	non small cell lung adenocarcinoma
C0007131	Non-Small Cell Lung Carcinoma	T191	Neoplastic Process	non small cell carcinoma of the lung
C0007131	Non-Small Cell Lung Carcinoma	T191	Neoplastic Process	non small cell adenocarcinoma of the lung
C0149925	Small cell carcinoma of lung	T191	Neoplastic Process	small cell lung carcinoma
C0149925	Small cell carcinoma of lung	T191	Neoplastic Process	small cell lung adenocarcinoma
C0149925	Small cell carcinoma of lung	T191	Neoplastic Process	small cell carcinoma of the lung
C0149925	Small cell carcinoma of lung	T191	Neoplastic Process	small cell adenocarcinoma of the lung
C1306460	Primary malignant neoplasm of lung	T191	Neoplastic Process	lung carcinoma
C1306460	Primary malignant neoplasm of lung	T191	Neoplastic Process	lung adenocarcinoma
C1306460	Primary malignant neoplasm of lung	T191	Neoplastic Process	carcinoma of the lung
C1306460	Primary malignant neoplasm of lung	T191	Neoplastic Process	adenocarcinoma of the lung

*CUI* Unified Medical Language System's Concept Unique Identifier

## Appendix

**Table 7:** Evaluation outcomes of the different implementations and datasets

				Named-entity recognition					Attribute relation detection	
Implementation	Set	Approach	Outcome	Lung cancer^a^		Non-small cell lung cancer^b^		Recurrence^c^	Recurrent lung cancer^d^	
				Present	Absent	Present	Absent	Present	Present	Absent
1	Development set (*n* = 30)	Relaxed	Detected	15	1	11	1	0		
cTAKES with UMLS 2011			Not detected	12	2	6	12	15		
		Strict	Detected	15	1	11	1			
			Not detected	8	6	6	12			
	Test set (*n* = 68)	Relaxed	Detected	35	0	29	1	0		
			Not detected	16	17	7	31	30		
		Strict	Detected	33	2	29	1			
			Not detected	7	26	2	36			
2	Development set (*n* = 30)	Relaxed	Detected	10	0	5	0	0		
cTAKES with UMLS 2016			Not detected	17	3	12	13	15		
		Strict	Detected	10	0	5	0			
			Not detected	13	7	12	13			
	Test set (*n* = 68)	Relaxed	Detected	29	0	18	1	0		
			Not detected	22	17	18	31	30		
		Strict	Detected	29	0	18	1			
			Not detected	11	28	13	36			
3	Development set (*n* = 30)	Relaxed	Detected	23	0	13	0	15		
cTAKES with UMLS 2016 and custom dictionary			Not detected	4	3	4	13	0		
		Strict	Detected	23	0	13	0			
			Not detected	0	7	4	13			
	Test set (*n* = 68)	Relaxed	Detected	36	0	22	1	30		
			Not detected	15	17	14	31	0		
		Strict	Detected	36	0	22	1			
			Not detected	4	28	9	36			
4	Development set (*n* = 30)	Relaxed	Detected	23	0	17	0	15	6	0
DIRECT cTAKES with UMLS 2016, custom dictionary, and post-processing			Not detected	4	3	0	13	0	0	10
		Strict	Detected	23	0	17	0			
			Not detected	0	7	0	13			
	Test set (*n* = 68)	Relaxed	Detected	36	0	28	1	29	9	0
			Not detected	15	17	8	31	1	3	18
		Strict	Detected	36	0	28	1			
			Not detected	4	28	3	36			

^a^SNOMED CT concept 93880001 | Primary malignant neoplasm of lung (disorder)

^b^SNOMED CT concept 254637007 | Non-small cell lung cancer (disorder)

^c^SNOMED CT concept 255227004 | Recurrent (qualifier value)

^d^Relationship between three SNOMED CT concepts: 93880001 | Primary malignant neoplasm of lung (disorder) |: 263502005 | Clinical course (attribute) | = 255227004 | Recurrent (qualifier value)

## Abbreviations

API: Application Programming Interface
cTAKES: Clinical Text Analysis and Knowledge Extraction System
DIRECT: Disease Information and Relationship ExtraCtion Tool
EHR: Electronic Health Record
MLP: Medical Language Processing
MRCM: SNOMED CT Machine Readable Concept Model
NLP: Natural Language Processing
UIMA: Unstructured Information Management Architecture
UMLS: Unified Medical Language System
